# Information Avoidance in Decision Making: Do the Reasons Vary by Age?

**DOI:** 10.1093/geroni/igaa057.1172

**Published:** 2020-12-16

**Authors:** Stephanie Deng, Julia Nolte, Corinna Loeckenhoff

**Affiliations:** 1 Cornell University, Rochester, Minnesota, United States; 2 Cornell University, Ithaca, New York, United States

## Abstract

Older adults make up the majority of the U.S. patient population and age differences in information avoidance have potential implications for their ability to participate in informed medical decision making. Meta-analytic evidence suggests that older adults seek less information before making a decision than younger adults do (Mata & Nunes, 2010). However, age differences in explicit information avoidance have yet to be quantified. We hypothesized that older adults would avoid decision-relevant information more strongly than younger adults do. We also examined the self-reported reasons for information avoidance and hypothesized that older adults would express more concern about unwanted information influencing their affect (Reed & Carstensen, 2012) and decision preferences (Mather, 2006), both of which are known predictors of information avoidance (Woolley & Risen, 2018). To test these assumptions, we conducted a pre-registered online study involving three different health-related decision scenarios. For each scenario, an adult lifespan sample (N=195, Mage=52.95, 50% female, 71% non-Hispanic White) chose to either receive or avoid information. Responses were highly correlated across scenarios and results were pooled into a single avoidance measure. Analyses indicated that concerns about consequences for decision preferences positively predicted decision avoidance (p<.001), whereas concerns about consequences for affect did not (p=.079). Contrary to predictions, older age was not significantly associated with information avoidance (p=.827). Further, self-reported concerns about the influence of unwanted information on affect and decision preferences were negatively associated with age (ps<.001). This suggests that interventions to foster pre-decisional information seeking should be tailored to the target age group.

